# CLIC1 Inhibition Attenuates Vascular Inflammation, Oxidative Stress, and Endothelial Injury

**DOI:** 10.1371/journal.pone.0166790

**Published:** 2016-11-18

**Authors:** Yingling Xu, Ji Zhu, Xiao Hu, Cui Wang, Dezhao Lu, Chenxue Gong, Jinhuan Yang, Lei Zong

**Affiliations:** 1 College of Life Science, Zhejiang Chinese Medical University, Hangzhou, China; 2 Clinical Laboratory, The Third Affiliated Hospital of Zhejiang Chinese Medical University, Hangzhou, China; University of Illinois at Chicago, UNITED STATES

## Abstract

Endothelial dysfunction, which includes endothelial oxidative damage and vascular inflammation, is a key initiating step in the pathogenesis of atherosclerosis (AS) and an independent risk factor for this disorder. Intracellular chloride channel 1 (CLIC1), a novel metamorphic protein, acts as a sensor of cell oxidation and is involved in inflammation. In this study, we hypothesize that CLIC1 plays an important role in AS. Apolipoprotein E-deficient mice were supplied with a normal diet or a high-fat and high-cholesterol diet for 8 weeks. Overexpressed CLIC1 was associated with the accelerated atherosclerotic plaque development, amplified oxidative stress, and in vivo release of inflammatory cytokines. We subsequently examined the underlying molecular mechanisms through in vitro experiments. Treatment of cultured human umbilical vein endothelial cells (HUVECs) with H_2_O_2_ induced endothelial oxidative damage and enhanced CLIC1 expression. Suppressing CLIC1 expression through gene knocked-out (CLIC1^−/−^) or using the specific inhibitor indanyloxyacetic acid-94 (IAA94) reduced ROS production, increased SOD enzyme activity, and significantly decreased MDA level. CLIC1^−/−^ HUVECs exhibited significantly reduced expression of TNF-α and IL-1β as well as ICAM-1 and VCAM-1 at the protein levels. In addition, H_2_O_2_ promoted CLIC1 translocation to the cell membrane and insertion into lipid membranes, whereas IAA94 inhibited CLIC1 membrane translocation induced by H_2_O_2_. By contrast, the majority of CLIC1 did not aggregate on the cell membrane in normal HUVECs, and this finding is consistent with the changes in cytoplasmic chloride ion concentration. This study demonstrates for the first time that CLIC1 is overexpressed during AS development both in vitro and in vivo and can regulate the accumulation of inflammatory cytokines and production of oxidative stress. Our results also highlight that deregulation of endothelial functions may be associated with the membrane translocation of CLIC1 and active chloride-selective ion channels in endothelial cells.

## Introduction

Atherosclerosis (AS) is the main pathological basis of cardiovascular diseases, which are the leading cause of morbidity and mortality worldwide [1]; AS presents complex pathogenesis involving endothelial dysfunction, smooth muscle cell proliferation, lipid infiltration, and inflammation. Endothelial dysfunction is a systemic disorder that represents a key early step in AS development [2]. Previous studies showed that endothelial dysfunction is an important mechanism responsible for AS. Hydrogen peroxide (H_2_O_2_), an intracellular secondary messenger in vascular remodeling, inflammation, and apoptosis, can effectively cause vascular endothelial dysfunction and thus promote AS [3, 4].

Oxidative stress is defined as the imbalance between the degree of oxidative stress and antioxidant defense capability, and this condition promotes reactive oxygen species (ROS) production. Decreased antioxidant enzyme activity and ROS scavenging ability can induce destruction of the antioxidant system. Consequently, overproduction of ROS and enhanced lipid peroxidation and malonic dialdehyde (MDA) production may contribute to oxidative stress in AS. Generally, vascular endothelial cells (ECs) are the ROS sources in vessel walls and participate in vessel pathology [5].

AS has been recognized as a chronic inflammatory disorder over the last 12 years [6, 7]. Inflammation is an integral component of AS from inception onwards and participates pivotally in all stages of AS from the lesion initiation to the progression and destabilization [8]. Adhesion of monocytes on vascular endothelium and subsequent transmigration into the arterial wall are characteristic features of inflammation phases in the pathogenesis of arteriosclerosis [6]. Although a normal endothelium maintains a non-adhesive surface, ECs secrete cell adhesion molecules (CAMs) in response to inflammatory mediators, such as TNF-α and IL-1β, which can promote the recruitment of monocytes across the endothelial barrier [9,10]. Moreover, ICAM-1 and VCAM-1 play important roles in aggravating AS [11].

Chloride intracellular channel 1 (CLIC1), a 241-amino acid protein that belongs to the CLIC family, is originally cloned based on its enhanced expression in activated human macrophages and is highly conserved among several species. CLIC1 shows a wide tissue and subcellular distribution in mammalian cells [12] and exists in a soluble form in the cytoplasm and nucleoplasm. In response to several stimuli, CLIC1 undergoes major structural changes and is inserted into lipid membranes to form a chloride-selective ion channel [13, 14]. Under oxidative stress, CLIC1 automatically transfers to the cell membrane and is inserted into lipid membranes [14–16]. CLIC1 is also involved in cell cycle regulation as well as cell proliferation and differentiation [17, 18].

Moreover, CLIC1 is overexpressed in tumors, such as hepatocellular carcinoma [19], gallbladder carcinoma [20], gastric carcinoma [21], and colorectal cancer [22, 23]. The N-terminal domain of CLIC1 contains a binding site for the reduced form of glutathione (GSH) [24]. Several lines of evidence indicate that CLIC1 acts as a sensor of cell oxidation [16, 25–26]. In a previous study, the truncated Aβ25–35 form increased CLIC1 chloride conductance in cortical microglia and induced microglial neurotoxicity; the CLIC1 ion channel blocker IAA-94 prevented cell injury and reduced free-radical generation. Furthermore, upon interaction with heat-inactivated Candida albicans cells, the proteomics of RAW 264.7 macrophages showed reduced CLIC1 expression which suppressed TNF-α and p-ERK levels through an anti-inflammatory response [27]. These data demonstrate the importance of CLIC1 in regulating macrophage function through its ion channel activity; hence, CLIC1 is a suitable target for the development of anti-inflammatory drugs [28].

CLIC1 plays an important role in AS pathogenesis. However, the relationship between CLIC1 overexpression and AS remains unclear. In this study, we investigated the effect of CLIC1 on vascular inflammation, oxidative stress, endothelial function, and atherogenesis in a mouse model of AS. The underlying molecular mechanism was also evaluated through cell culture experiments.

## Materials and Methods

### Animals and treatment protocol

Apolipoprotein E-deficient (ApoE^−/−^) male mice aged 8 weeks (Cavens Lab, China) were used for the experiment. The animals were housed in a 22°C room with a 12:12-h light/dark cycle. Mice were randomly divided into two groups. The control mice were fed with a normal chow diet. The AS model mice were provided with a high-fat and high-cholesterol (HFHC) diet consisting of 10% fat, 4% milk, 2% cholesterol, and 0.5% sodium cholate (Cavens Lab, China) for 8 weeks. All the mice were maintained with access to water ad libitum. Body weights were measured every week. After the treatment, the mice were sacrificed by carotid artery exsanguination under adequate anesthesia (1% sodium pentobarbital, 50 mg/kg via i.p. injection, no limb reaction after pressure). Blood was immediately obtained for analyses. The abdomen and thoracic cavities were opened, and tissue samples (aorta and liver) were isolated. This study was performed in strict accordance with the recommendation in the Guide for the Care and Use of Laboratory Animals of the National Institutes of Health. The protocol was approved by the Committee on the Ethics of Animal Experiments of Zhejiang Chinese Medical University.

### Reagents

Indanyloxyacetic acid-94 (IAA-94) was purchased from Sigma (MO, USA). Anti-ICAM-1 and VCAM-1 antibodies were obtained from ImmunoWay Biotechnologies (California, USA). Anti-CLIC1 antibody was acquired from Santa Cruz Biotechnologies (sc-81873, CA, USA). Anti-Na^+^/K^+^-ATPase antibody was acquired from SanYing Biotechnology (14418-1-AP, Wuhan, China). Goat Anti-Mouse IgG (CW0102) and Goat Anti-Rabbit IgG (CW0103) were acquired from CWBIO (Beijing, China). FITC Goat Anti-Mouse IgG was acquired from EarthOx (E031210-01, CA, USA). Hydrogen peroxide solution (H_2_O_2_) was provided by Sigma. Chemicals and reagents of analytical grade were obtained from Sigma, TOYOBO, or Invitrogen.

### Biochemical assays

Total serum cholesterol (TC), triglyceride (TG), low-density lipoprotein (LDL), and high-density lipoprotein (HDL) levels in plasma were measured using an automatic biochemical analyzer (Hitachi, Japan) according to the manufacturer’s instructions. LDH activity in the plasma was also determined using the analyzer.

### Determination of oxidant/anti-oxidant status

#### Detection of SOD activity

SOD activity was detected based on the inhibition of nitro blue tetrazolium reduction using the xanthine/xanthine oxidase system as a superoxide generator. Absorbance was determined with a Microplate reader (Molecular Devices, USA) at 560 nm. Enzyme activity assays were performed in triplicate, and the results were averaged and expressed as U/mg of sample protein.

#### MDA levels in HUVECs and tissues

Lipid peroxidation levels in HUVECs and liver were determined based on the concentration of thiobarbituric acid-reactive substances, with the MDA content as the index of lipid peroxidation. Absorbance was determined using a Microplate reader at 532 nm. MDA concentration was calculated using the absorbance coefficient according to the instruction and expressed as nmol/mg of protein.

#### GSH and GSSG levels in tissues

GSH concentration in tissues was measured with a GSH and GSSG Assay Kit (Beyotime, China) by using 5,5′-dithiobis-(2-nitrobenzoic acid) recycling method. The results were expressed as μM and compared with that of the standard solution of GSSG. In addition, GSH was calculated as 2× GSSG. Absorbances were monitored using the microplate reader at 412 nm.

### Assessment of AS

The entire aorta, extending from the aortic arch to the iliac bifurcation, was removed, cleaned out of adventitia, and fixed with formalin. The aortic arch was opened longitudinally, sliced horizontally into 4μm-thick sections, and placed on glass slides. The aortas obtained from ApoE^−/−^ mice were stained with hematoxylin and eosin (H&E) to evaluate atherosclerotic lesions in the aortic root. The lesion area was quantified on every fourth section, and the average was determined. The samples were photographed using a Nikon Ti-S Eclipse. The lesion areas were quantified using IMAGEPRO PLUS software (Media Cybernetics, USA).

### Immunohistochemistry

The aortic arch was sliced into horizontal sections, fixed with 10% formalin overnight, embedded in paraffin, and cut into 4μm-thick sections. The aortic arch sections were immunostained with antibodies against CLIC1 (Santa Cruz; working dilution 1:50) to investigate whether CLIC1 was actively expressed in aortic lesions. After quenching of endogenous peroxidase activity and incubation with 10% goat serum blocking buffer and primary antibodies, the specimens were incubated with biotin-labeled goat anti-mouse secondary antibody. The specimens were added with horseradish peroxidase (HRP)-conjugated streptavidin, and positive staining area was detected by 3, 3-diaminobenzidine. Three independent observers evaluated the immunohistochemical results.

### Cell culture

HUVECs were purchased from Shanghai Academy of Life Sciences (Shanghai, China) and cultured in DMEM (Hycolon, USA) supplemented with 10% fetal bovine serum (BI, Israel) at 37°C under a humidified atmosphere of 5% CO_2_ and 95% air.

### Gene knockdown of CLIC1 in vitro

The CLIC1 gene expression was knocked down in HUVECs by using the CRISPR/Cas9 binary vector lentivirus gene knockout technology as described previously [29]. CLICl sgRNA (*GCACTTCAGTGCCATACAGC*) was designed according to GenBank, inserted into the lentiviral vector with an appropriate restriction enzyme, and lentiviruses containing the desired sequence was packaged and purified by Shanghai Genechem. Pretreatment of cells for 4h with complete medium containing 5 μg / ml Polybrene was performed before lentivirus infection, and then lentiviral particles were thawed at room temperature, mixed gently before use. The virus suspension was added to cultured cells, shook gently to mix it, then incubated overnight, and replaced with normal culture medium the next day. Because the CAS9 + sg RNA viruses dual system needs two steps, CAS9 lentiviruses were used for the first infection. After Puromycin selection (0.5μg / ml for 48 h), surviving cells with the stable expression of CAS9 were then reinfected with lentiviruses containing the sg RNA. Stable clones were established by G418 screening (200μg / ml for 12 days), and fresh medium containing G418 was replaced every 3–4 days until resistant colonies can be identified. Knockdown efficiency in the cells was verified by Western blot analysis, and almost 100% of the protein expression was lost.

### Measurement of intracellular ROS generation

ROS generated in HUVECs was measured using 10 μmol/L fluorescent probes, 2′, 7′-dichloro-fluorescein diacetate (DCF-DA) (Beyotime, China), as described. HUVECs were treated with DCFH-DA for 30 min at 37°C and immediately washed three times with 1 mL of PBS. Similar to FITC, a multifunctional fluorescence microscope (Leica DMi 8, Germany) was used at 485nm excitation and 525nm emission wavelengths to capture images with LAS X software.

### Measurement of intracellular Cl^−^

After HUVECs were digested and transferred into separate suitable containers in the same density, the cells were then incubated with 5 mM *N*-(ethoxycarbonylmethyl)-6-methoxyquinolinium bromide (MQAE, molecular probes) for 30 min at room temperature in Krebs-HEPES solution (20mM HEPES, 128mM NaCl, 2.5mM KCl, 2.7mM CaCl_2_, 1mM MgCl_2_, and 16mM glucose; pH7.4). The incubated cells were planted back into the 96-well cell plate for imaging with Leica DMi 8, and the fluorescence was read using a fluorescence microplate reader.

### Quantification of released TNF-α and IL-1β

TNF-α and IL-1β in animal serum and HUVECs were determined using a mouse TNF-α and IL-1β enzyme-linked immunosorbent assay kit (4A Biotech, China) and human ELISA kit (4A Biotech, China), respectively, according to the manufacturer’s protocol.

### Analysis of gene expression by quantitative real-time PCR

Total RNA was isolated from mouse aortas and HUVECs. RNA yields were evaluated using UV-Vis Spectrophotometer Q5000 (Quawell, USA), and RNA samples with an A260/A280 ratio exceeding 1.8 were selected for subsequent experiments. Qualitative PCR was performed using ReverTra Ace qPCR RT kit (TOYOBO, Japan). Tubes containing the mixture were placed in a StepOnePlus Real-Time PCR system (AB, USA). The reaction comprised a reverse-transcription of an initial denaturation step at 95°C for 10 min, followed by 40 cycles at 95°C for 20 s, 59°C for 30 s, and 68°C for 30 s, and a final extension step at 72°C for 10 min [30]. The primers used in this study were as follows [17, 30]:

Human Beta actin forward primer: *5′-ACCAACTGGGACGACATGGAG-3′*;

reverse primer: *5′-GTGAGGATCTTCATGAGGTAGTC-3′*;

Human CLIC1 forward primer: *5′-AATCAAACCCAGCACTCAATG-3′*;

reverse primer: *5′-CAGCACTGGTTTCATCCACTT -3′*;

Mouse CLIC1 forward primer: *5′-CCCTGAGTCCAACACCTCG-3′*;

reverse primer: *5′-GCGCTGGTTTCATCCACTTC-3′*;

Mouse GAPDH forward primer: *5′-GGTGAAGGTCGGTGTGAACG-3′*;

reverse primer: *5′-CTCGCTCCTGGAAGATGGTG-3′*.

The mRNA expression levels of the target genes in each sample were expressed as a value relative to that of the control (ΔΔCt).

### Immunofluorescence assay

HUVECs cultured on covered glass chamber slides were fixed in 4% paraformaldehyde at room temperature for 15 min and permeabilized with 0.2% Triton X-100 in PBS for 15 min. After blocking with goat serum working solution for 0.5–1 h at 37°C, the cells were incubated with mouse monoclonal anti-CLIC1 (1:50 dilution) at 37°C for 2 h; FITC-conjugated goat anti-mouse IgG (1:400 dilution) was subsequently added, and the slides were incubated at 37°C for 1 h in the dark. The cells were washed three times with PBS, and DAPI was added for nuclear staining. The cells were observed under the Leica DMi 8 fluorescence microscope.

### Western blot analysis

Total protein, as well as membrane protein from the cells and aortas, was extracted by Membrane and Cytosol Protein Extraction Kit (P0033, Beyotime, China). According to the instructions, the cells were disrupted by homogenization, centrifuged at low speed to remove precipitates including the nuclei and unbroken cells. Then the supernatant was centrifuged at 14000g to separate the cell membrane precipitate and supernatant containing cytoplasmic protein, and the membrane protein was dissolved by membrane protein extraction reagent B. Equal amounts of protein were separated on 8% SDS–polyacrylamide gels and transferred onto polyvinylidene fluoride membranes by using a semi-dry transfer blotting system (Pyxis, China). The membranes were blocked with 5% skim milk for 2 h, incubated at 4°C overnight with primary antibodies, including antibodies against CLIC1, ICAM-1, VCAM-1, Na^+^/K^+^-ATPase, and β-actin, and incubated for 2 h with the HRP-conjugated secondary antibody. The blots were visualized using ECL-plus detection system (Clinx Science Instruments, China). Densitometric analysis of the bands was performed using the supplementary software.

### Statistical analysis

All results were expressed as mean ± SEM. Statistical analysis was assessed by GraphPad Prism 5.00 (San Diego, CA, USA). Comparisons between groups were made using the Student’s unpaired t-test or one-way ANOVA followed by the Tukey multiple comparison test. A p-value <0.05 was considered significant. All data were obtained from at least three independent experiments.

## Results

### 1 Cardio-metabolic effects of high-fat diet

Eight-week-old ApoE^−/−^ mice were fed with an HFHC diet for 8 weeks to build an AS model and detect cardiovascular indicators, including body weight as well as serum lipid and lactate dehydrogenase (LDH) levels. The body weight of the mice significantly increased after the 8 weeks of the cholesterol-rich diet (Table 1). The lipid levels were higher in the treated mice than those of the control mice fed with a normal chow diet. HFHC-treated ApoE^−/−^ animals displayed significantly elevated TC, TG, HDL, and LDL levels (Table 1). The atherosclerotic mice displayed changes in LDH levels compared to those of mice in the ApoE^−/−^ Cont group (Table 1). Hence, a mouse model of AS was successfully constructed and used for subsequent detection.

**Table 1:** cardio-metabolic effects of high-fat diet in ApoE^-/-^ mice ($\bar{\text{x}}\pm \text{s}$, n = 6).

Group/Biochemical Assays	ApoE^-/-^ Cont	ApoE^-/-^ AS
Body weights (g)	28.10±0.17	30.97±0.09*
Triglyceride (mg/dL)	108.02±6.20	229.33±10.63*
Total cholesterol (mg/dL)	750.20±53.75	1536.36±125.68*
High-density lipoprotein (mg/dL)	80.82±5.03	130.32±3.09*
Low-density lipoprotein (mg/dL)	544.09±47.56	1057.62±92.03*
Lactate dehydrogenase (IU/L)	413.04±60.07	659.68±166.62*

ApoE^-/-^ mice were fed an HFHC diet or a normal diet respectively for 8 weeks. To detect cardio-metabolic effects, body weight, serum lipid concentrations, and Lactate dehydrogenase were examined. The shown data represent the Mean ± SEM (n = 6, *p<0.05 versus control).

### 2 Atherosclerotic plaque area

**Fig 1:**
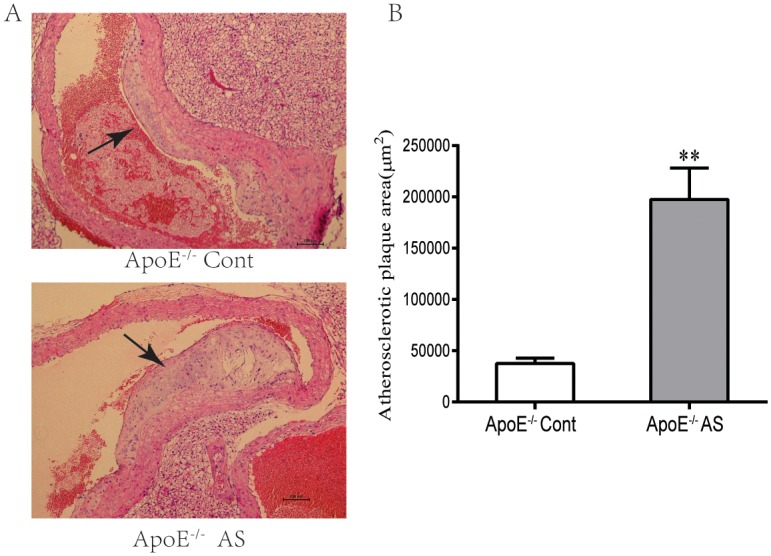
Atherosclerotic plaques in isolated aortic segments of normal or cholesterol-rich diet-treated ApoE^-/-^ mice after 8 weeks. **(A)** Representative histological cross-sections of the aortic root stained with H&E displayed atherosclerotic plaque development. Magnification 20×, bar 100μm. (**B)** Quantitative analysis of atherosclerotic lesion formation indicated as plaque areas (mean ± SEM, n = 6, *p<0.05 versus ApoE^-/-^ Cont).

H&E-stained sections of the aortic arches were observed to identify direct signs of AS. The area of the aortic atherosclerotic plaque was also examined. The ApoE^−/−^ mouse model developed spontaneous atherosclerotic lesions even when provided with a normal diet [31]. As shown in Fig 1A, spontaneous atherosclerotic lesions were formed in the ApoE^−/−^ mice given with normal or high-fat diets for 8 weeks. However, the area of the atherosclerotic lesions in the high-energy diet fed ApoE^−/−^ mice is four-fold higher than that in the ApoE^−/−^ mice fed with a normal diet (Fig 1B). These results demonstrated that the formation of plaques significantly differed between the two groups of ApoE^−/−^ mice treated with different diets.

### 3 Oxidative stress in vivo

**Fig 2:**
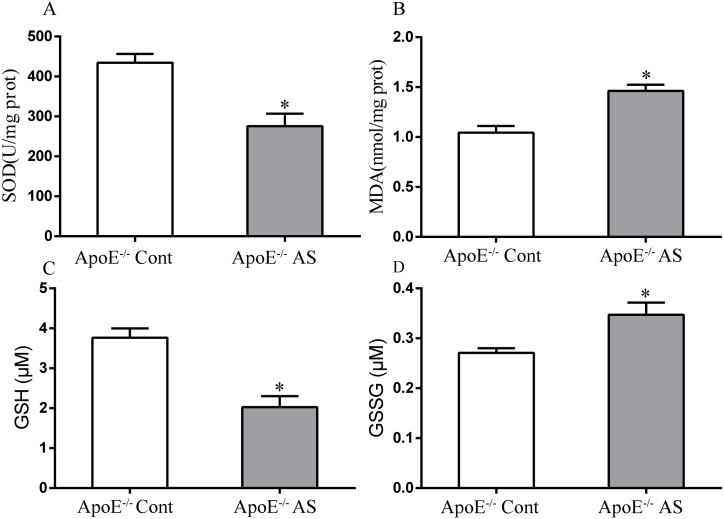
SOD activity (A) and MDA (B), GSH (C), GSSG (D) levels in liver tissues of atherosclerotic mice (n = 6). Each value represents the mean±SEM. *P < 0.05 versus control. SOD: superoxide dismutase; MDA: malondialdehyde; GSH: glutathione, GSSG: oxidized glutathione.

Oxidative stress is caused by damage to antioxidant defense and production of free radical; this type of stress plays a critical role in endothelial dysfunction and AS [32, 33]. In the present study, the systemic redox status in the whole body was estimated by detecting the antioxidant capacity and oxidant production in the liver. As the site for cholesterol metabolism, liver contains a high GSH content and is the major organ involved in its synthesis; GSH depletion is associated with the increased levels of lipid peroxides in the liver [34, 35]. Lipid peroxidation (MDA) and GSH levels are shown in Fig 2B and 2C, respectively. Generally, the atherogenic mice displayed significantly higher MDA levels (1.46 ±0.06 vs. 1.05 ± 0.07nmol/mg) (P <0.05), lower GSH levels (3.77 ±0.23 vs. 2.03 ±0.07μM) (P <0.05), and slightly higher GSSG levels than those of the controls. In addition, SOD is an antioxidant enzyme that rapidly dismutates O^2–^ into H_2_O_2_. The SOD activity of the antioxidant enzymes was significantly reduced in the atherosclerotic mice (20.76 ± 2.58 vs. 4.11 ±0.05 U/mg) (P <0.05), similar to the patterns of lipid peroxidation. The high MDA level is consistent with the increasing lipid levels in plasma, as shown by the lipid profile test (Table 1).

### 4 Expression of inflammatory cytokines in vivo

**Fig 3:**
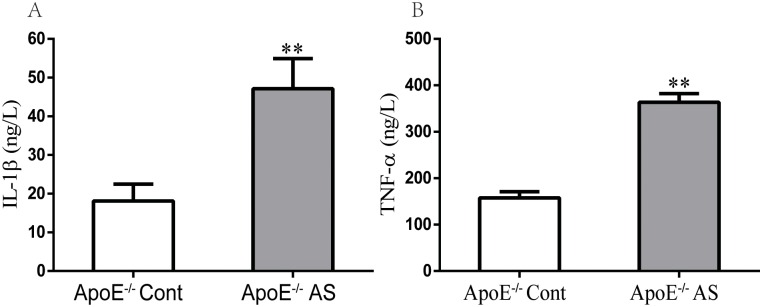
Expression of vascular pro-inflammatory markers in plasma from mice fed an HCHF or a standard chow diet. The levels of pro-inflammatory cytokines, IL-1β (**A**) and TNF-α (**B**), were determined by ELISA. The values are mean ± SEM of n = 6, **P < 0.01 versus control.

Inflammation and adhesion of mononuclear cells to the vascular wall are critical for AS initiation and development [36]. Inflammatory cytokines are important in the formation of lesional plaques and affect the entire atherosclerotic vessel [37]. Pro-inflammatory cytokines, namely TNF-α and IL-1β, play an important role in the destruction of vascular homeostasis in AS; their corresponding levels significantly increased in the plasma of ApoE^−/−^ mice fed with the HCHF diet (Fig 3).

### 5 CLIC1 is highly expressed in lesional plaques

**Fig 4:**
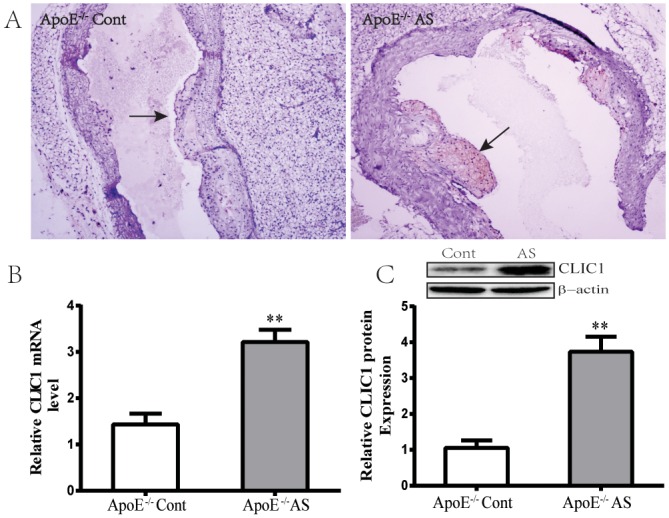
Overexpression of CLIC1 in lesional plaques and aortic arches. **(A)** CLIC1 in the atherosclerotic plaques was detected by immunohistochemistry using specific antibodies for CLIC1. Respective stainings at magnification 20× are shown. (**B)** CLIC1 mRNA content was quantified by qPCR. GAPDH was used as an internal control. Data are mean ± SEM of n = 6 animals per group. Representative results of at least three animals per group are shown. (**C)** CLIC1 protein expression was determined by the Western blot technique. Representative original blots are shown below the densitometric quantification for three animals per group. **P < 0.01 versus ApoE^-/-^ Cont group.

CLIC1 is an executor of cell oxidation [25]. We, therefore, hypothesized that CLIC1 is involved in the lesion formation. To investigate whether CLIC1 exists in pathological arteries, we detected the expression of CLIC1 protein in lesional plaques by using immunohistochemical staining. Representative photographs of CLIC1 antibody immunostained sections were shown in Fig 4A. The result showed that the atherogenic diet induced a significant increase in the CLIC1 level compared with the normal diet. CLIC1 expression was then assessed using the aortic tissue of ApoE^−/−^AS mice by using qPCR (Fig 4B) and Western blot analyses (Fig 4C). CLIC1 was overexpressed in the aortic tissue of ApoE^−/−^ mice that received the fat-rich diet for 8 weeks. These findings indicated that CLIC1 expression is associated with the presence of atherosclerotic plaques. Thus, we aimed to further clarify the role of CLIC1 in AS through in vitro experiments.

### 6 Effects of H_2_O_2_ on CLIC1 expression

**Fig 5:**
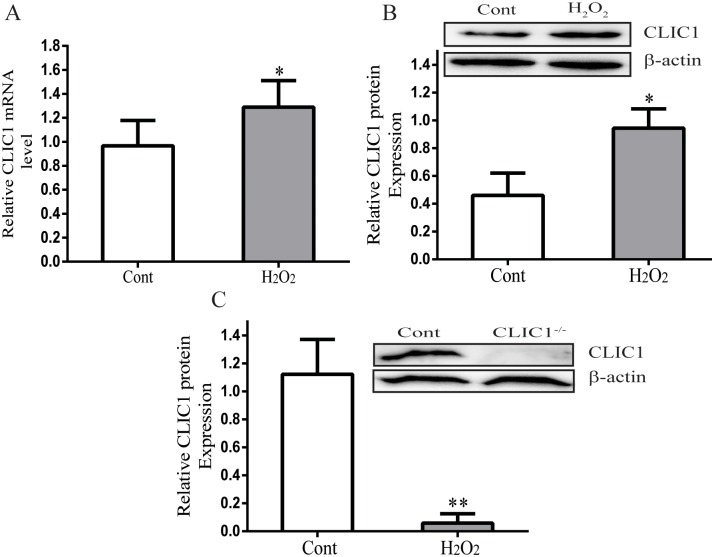
Effects of hydrogen peroxide on CLIC1 expression in HUVECs. **(A) and (B)** A significant increased CLIC1 mRNA and protein expression was observed in 0.9mM H_2_O_2_ exposed HUVECs for 12 h. (**C)** CLIC1 was almost not detectable after the CLIC1 knockout. β-actin was used as a loading control. Quantitative data were presented as mean ± SEM of three replicate experiments. The significance of differences with respect to untreated cells was calculated using Student’s unpaired t-test. *P < 0.05, **P < 0.01 versus Cont group.

CLIC1 expression at the mRNA and protein levels was measured from cell lysates after incubation of HUVECs with H_2_O_2_ (Fig 5A and 5B). Consistent with the in vivo findings, H_2_O_2_ incubation significantly enhanced CLIC1 expression. To detect the role of CLIC1 in endothelial injury, we constructed CLIC1^-/-^ HUVECs. CLIC1 knockout dramatically reduced CLIC1 expression as shown in Fig 5C.

### 7 CLIC1 regulates oxidative stress in vitro

**Fig 6:**
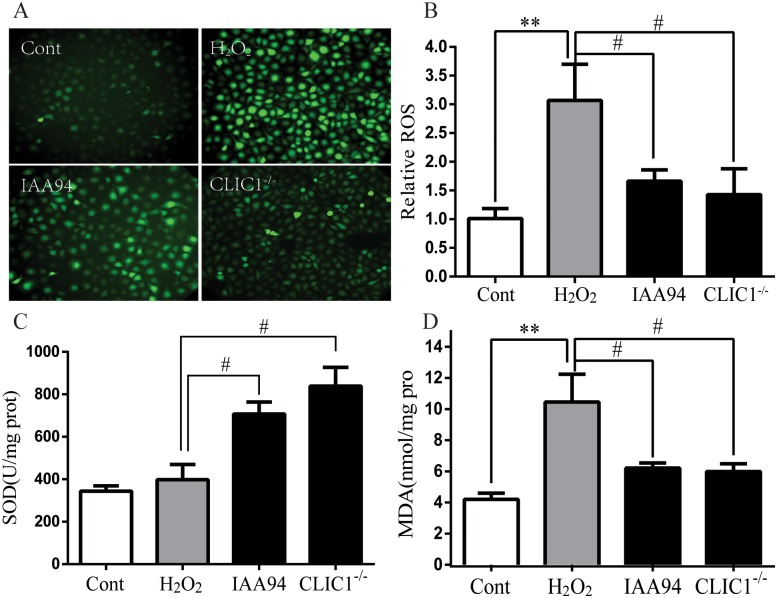
Inhibition of CLIC1 suppresses oxidative stress. **Cont group:** HUVECs with normal culture medium for 12h. **H**_**2**_**O**_**2**_ **group:** HUVECs with H_2_O_2_ exposure for 12h. **IAA94 group:** HUVECs with 40μM IAA94 pretreatment for 1 h and then H_2_O_2_ exposure for 12h. **CLIC1**^**-/-**^ **group:** CLIC1^-/-^ HUVECs with H_2_O_2_ exposure for 12h. (**A) and (B)** intracellular ROS production was measured by DCF fluorescence microscopy (A), and the fluorescence intensity was quantified (B). (**C)** and **(D)** SOD activity and MDA production were detected as the products of oxidative stress. IAA94 blunted the H_2_O_2_-induced decrease of SOD activity and increase of MDA. CLIC1^-/-^ HUVECs displayed a higher SOD activity and a lower MDA level. The data was shown as mean ± SEM of n = 6. **P < 0.01 versus Cont group, ^#^P < 0.05 versus H_2_O_2_ group.

We evaluated the effects of CLIC1 inhibition on oxidative stress in ECs. The results showed that ROS generation significantly increased after incubation with H_2_O_2_ alone, whereas IAA-94 significantly reduced DCF fluorescence in the cells exposed to H_2_O_2_. This finding indicated that CLIC1 inhibition suppressed oxidative stress and significantly reduced ROS production (Fig 6A and 6B). Moreover, incubation of CLIC1^−/−^ HUVECs with H_2_O_2_ alone did not affect the formation of basal cellular ROS (Fig 6A and 6B). Consistent with the pattern above, H_2_O_2_-induced decrease in SOD oxidase activity was inhibited by IAA-94 in HUVECs. Similar to the effect of IAA-94 incubation, CLIC1 deficiency significantly upregulated SOD activity and downregulated MDA expression (Fig 6C and 6D). These results suggested that oxidative stress was abated by inhibition of CLIC1 by IAA-94 or gene deficiency. These results demonstrate that CLIC1 overexpression may play an important role in oxidative stress of atherogenesis.

### 8 CLIC1 inhibition suppresses adhesion and inflammation

**Fig 7:**
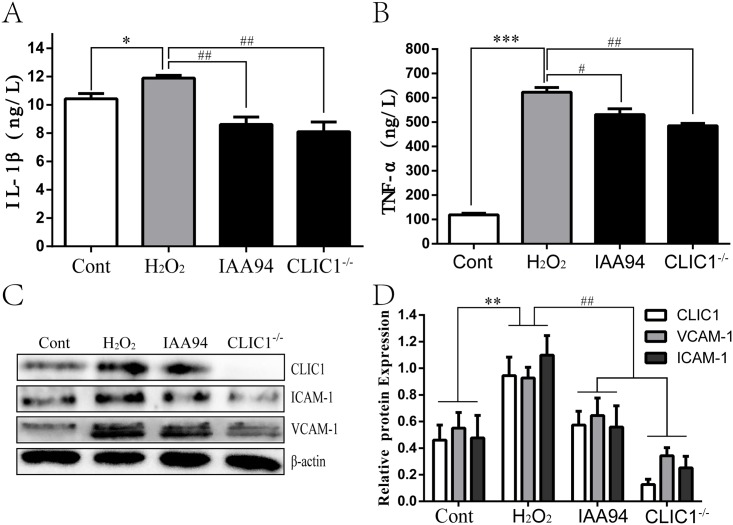
CLIC1 promotes adhesion of monocytes to and inflammation of ECs under oxidative stress. **Cont group:** HUVECs with normal culture medium for 12h. **H**_**2**_**O**_**2**_ **group:** HUVECs with H_2_O_2_ exposure for 12h. **IAA94 group:** HUVECs with 40μM IAA94 pretreatment for 1 h and then H_2_O_2_ exposure for 12h. **CLIC1**^**-/-**^ **group:** CLIC1^-/-^ HUVECs with H_2_O_2_ exposure for 12h. (**A)** and **(B)** The pro-inflammatory cytokines IL-1β (A) and TNF-α (B) levels determined by ELISA both were increased after H_2_O_2_ exposure, whereas IAA94 and CLIC1 deficiency markedly obstructed their increase. (**C)** and **(D)** IAA94 and CLIC1 deficiency obviously reduced ICAM-1 and VCAM-1 relative expression levels at the protein level. *P < 0.05, **P < 0.01 versus Cont group, ^#^P < 0.05, ^##^P < 0.01 versus H_2_O_2_ group. Error bars represent SD of three replicate experiments.

We assessed the role of CLIC1 in pro-inflammation by examining the expression of pro-inflammatory cytokines (TNF-α and IL-1β) in ECs and aortic tissues. Moreover, adhesion molecules (ICAM-1 and VCAM-1), induced by TNF-α and IL-1β, which can promote adherence of monocytes in AS development, were also measured in ECs [26, 37]. To further study whether CLIC1 could result in a pro-inflammatory response, IAA-94 and CLIC1^-/-^ HUVECs were adopted. The results showed that CLIC1 inhibition remarkably reduced IL-1β and TNF-α expression enhanced by treatment with H_2_O_2_ alone (Fig 7A and 7B). The expression levels of ICAM-1 and VCAM-1 were highly elevated in HUVECs by H_2_O_2_ and down-regulated in CLIC1^−/−^ cells (Fig 7C and 7D). These findings indicate that CLIC1 can exacerbate adhesion and inflammation of ECs.

### 9 H_2_O_2_ exposure promotes CLIC1 membrane translocation

**Fig 8:**
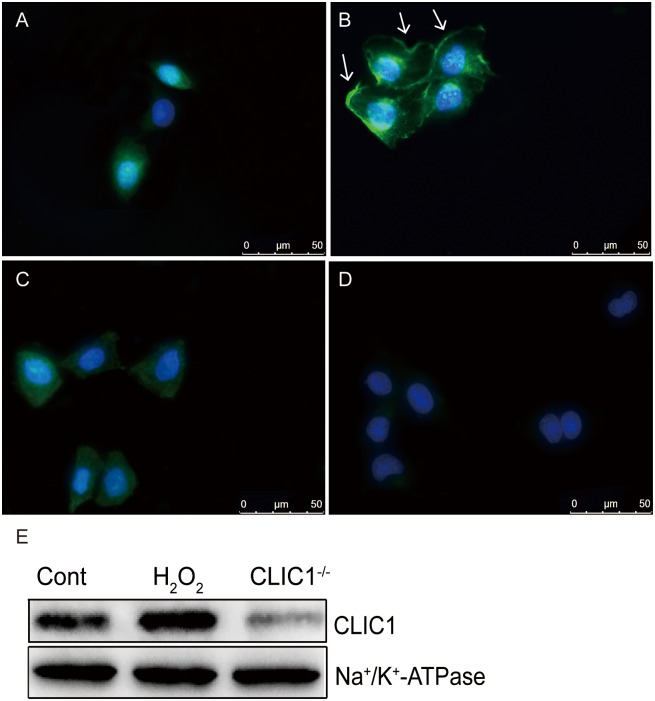
CLIC1 protein translocates to the cell membrane during H_2_O_2_ exposure as shown by immunofluorescence analysis in HUVECs. The cells were fixed and immunostained with CLIC1 mouse monoclonal antibody and with a corresponding secondary fluorescence-labeled antibody to observe CLIC1 translocation (green), nuclear (blue), and merged images were shown. **(A)** CLIC1 distribution in normal HUVECs. **(B)** Stimulation with 0.9mM H_2_O_2_ for 12h resulted in the translocation of upregulated CLIC1 into the cell membrane (arrows) and the reduction of cytoplasmic fluorescence. **(C)** Cytoplasmic localization of CLIC1 was markedly reduced in cells pretreated with blocker IAA94. **(D)** The fluorescence was quite weak in CLIC1^-/-^ cells. Images are representatives of three independent experiments. Scale bars 50μm, Magnification 40×. **(E)** WB of isolated membrane fractions after H_2_O_2_ stimulation showed elevated CLIC1 proteins in the membrane fraction. Na^+^/K^+^-ATPase was used as a loading control.

A moderate nuclear and cytoplasmic fluorescence was observed in the control cells. As shown in Fig 8, in H_2_O_2_-stimulated cells, evidently increased fluorescence was predominantly localized to the cell membrane. Upon H_2_O_2_ stimulation of the IAA-94-treated cells, fluorescence intensity reduced, and a substantial part of the fluorescence remained in the cytoplasmic compartment. In addition, a weak fluorescence was observed in CLIC1^−/−^ HUVECs. This result indicated that a high degree of CLIC1 membrane translocation occurred and may have regulated oxidative stress and inflammation under the oxidative stimulus.

### 10 Intracellular Cl^−^ concentration

**Fig 9:**
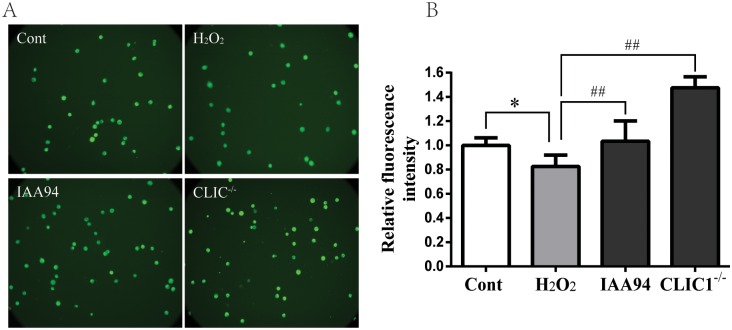
Changes of intracellular chloride concentration in HUVECs. **(A) The** fluorescence signal in non-treated cells was strong after staining with MQAE dye. Stimulation of the cells with 0.9mM H_2_O_2_ for 12h resulted in a complete quenching of fluorescence for the high concentration of Cl^−^ bound to and suppressed the MQAE dye. In the cells pretreated with the blocker IAA94, the fluorescence was markedly brighter than the cells of the H_2_O_2_ group. In CLIC1^-/-^ cells, the fluorescence was quite close to Cont group in intensity. (**B)** Fluorescent intensity was quantified accurately. *P < 0.05 versus normal group, ^##^ P < 0.01 versus H_2_O_2_ group. Error bars represent SD of three replicate experiments. Images are representatives of three independent experiments. Magnification 20×.

Intracellular Cl^−^ concentration was measured in HUVECs by using the Cl^−^ quenching dye MQAE as a fluorescent Cl^−^ indicator [38]. Higher fluorescence intensity, meaning of a low Cl^−^ concentration, was observed in the cells whose soluble CLIC1 channels were inhibited by IAA-94, and the Cl^−^ concentration was even lower in the CLIC^−/−^ HUVECs (Fig 9).

## Discussion

CLIC1 is a potential prognostic marker for some malignant tumors, and elevated CLIC1 expression is correlated with poor prognosis [39]. Following an increase in expression, CLIC1 will translocate to the membrane in a cytosolic oxidative state [16, 40, 41]; this phenomenon corresponds to a peak of ROS during G1-S transition [42, 43]. CLIC1 ablation impaired the capacity of phagosomal proteolysis and reduced ROS through its ion channel activity in CLIC1^−/−^ macrophages, and similarly, CLIC1^−/−^ mice were protected from K/BxN arthritis, both suggesting that CLIC1 is a suitable target for anti-inflammation [28]. Considering that CLIC1 is a novel metamorphic protein [44] involved in the regulation of oxidative stress responses and inflammation, we investigated its role in AS, whose insertion into the membrane to form active ion channel alteration could lead to endothelial dysfunction.

This study shows for the first time that CLIC1 expression is enhanced in the atherosclerotic ApoE^−/−^ mouse model, consistent with the observations of CLIC1 upregulation and endothelial cell dysfunction after exposure to H_2_O_2_ in vitro. First, in the animal experiment, the ApoE^−/−^ mice fed with a high-fat diet showed higher weights and displayed significantly elevated serum cholesterol, TG, HDL, LDL, and LDH levels compared with the control mice. H&E staining revealed that the HFHC-treated mice developed numerous large aortic atherosclerotic plaques, indicating that an atherosclerotic model was successfully built. Interestingly, immunohistochemical analysis showed that the formation of atherosclerotic lesions in the aortic arch was accompanied with CLIC1 overexpression. Moreover, a high level of oxidative damage and inflammatory markers in the atherosclerotic mice was detected.

We adopted the CRISPR/Cas9 binary vector lentivirus gene knockout technology to construct CLIC1^−/−^ HUVEC and used the CLIC1 specific blocker (IAA-94) to inhibit CLIC1 expression. We then evaluated the effects of CLIC1 downregulation on oxidative damage of ECs and the expression of pro-inflammatory cytokines (IL-1β and TNF-α) and adhesion molecules (ICAM-1 and VCAM-1) in the ECs with vascular inflammatory reaction. We also studied whether membrane translocation could occur. Studies have shown that knockout of CLIC1 improved SOD activity and suppressed the expression of ROS and MDA; hence, CLIC1 plays a crucial role in maintaining the balance between the degree of cellular oxidative stress and antioxidant defense capability.

Considering that oxidation can promote the insertion of CLIC1 into the membrane to form chloride ion channels [15], we investigated whether CLIC1 membrane translocation contributed to the increased oxidative injury to ECs and enhanced the expression of pro-inflammatory factors. CLIC1 was translocated to the cell membrane under oxidative stress induced by H_2_O_2_, and the Cl^−^ concentration of the H_2_O_2_-treated cells was higher than that of the cells whose CLIC1 was inhibited. These findings indicated a correlation between the membrane translocation of CLIC1 and exacerbation of endothelial cell oxidative damage as well as vascular inflammation during AS.

The atherosclerotic model used in this study (ApoE^−/−^) is well-established, as described in numerous publications [45]. Feeding ApoE^−/−^ mice with a high-energy diet leads to the development of atherosclerotic plaques and endothelial dysfunction [31]. ApoE^−/−^ atherosclerotic mice exhibited the advanced lesion development with a pronounced oxidative stress and inflammatory accumulation accompanied by the excessive increase in CLIC1. However, what role CLIC1 plays is still unclear. Furthermore, we investigated the relationship between CLIC1 and endothelial dysfunction as the earliest event in atherogenesis [37].

Endothelial dysfunction can also serve as an independent indicator to predict future cardiovascular events. Healthy vascular ECs exhibit unique regulatory functions by affecting angiogenesis, vascular tone, blood clotting, and vascular inflammation [46]. Endothelial cell dysfunction, mainly including decreased function of anticoagulant, anti-cell adhesion, and antioxidant, causes a series of pathological changes that lead to AS. ROS, a major risk for endothelial dysfunction, control numerous signaling pathways relevant to AS, mainly through inducing oxidative damage and inflammation in ECs [47].

Overproduction of ROS is a major feature of several pathological states; many cancers and neurodegenerative diseases are accompanied by the altered redox balance caused by dysregulation of nicotinamide adenine dinucleotide phosphate hydrogen (NADPH) oxidase. Many reports indicated that CLIC1 is highly expressed in diseases that involved oxidative stress. Oxidative stress also leads to rapid GSH depletion, reduced antioxidant enzyme, lipid peroxidation, and DNA damages [48]. The N-terminal domain of CLIC1 bears a binding site for the reduced form of GSH, and the protein undergoes drastic changes upon oxidation. GSH detachment from the CLIC1 binding site consequently rearranges the amino terminus of the protein and probably exposes the hydrophobic region that interacts with the cell membrane [16, 25]. The expression of GSH was reduced in the in vivo experiment and is thus considered as a specific indicator of oxidative stress [49]. The result showed that CLIC1 acted as both a secondary messenger and an executor of cell oxidation [25] by blocking GSH synthesis to impair the antioxidant defense. The reduced GSH activity may disrupt the ability of tissues to counteract the fast generation of superoxide anions (O_2_^−^) or protect the cells from reactive free radicals and peroxides [50].

Some studies have shown that AS is a chronic inflammatory disease associated with endothelial dysfunction [51]. CLIC1 ablation markedly attenuated the expression of TNF-α and IL-1β, two most important inflammatory cytokines that mediate systemic inflammation [52–54], which was consistent with the result of the in vivo experiment. When ECs undergo inflammatory activation, the increased expression of adhesion molecules plays important roles in blood monocyte recruitment to the arterial intima [55]. ICAM-1 and VCAM-1 were subsequently measured. These data suggested that CLIC1 accelerates vascular inflammation by enhancing the expression of TNF-α and IL-1β, which resulted in increased ICAM-1 and VCAM-1 levels to facilitate firm adhesion of leukocytes onto ECs during the prophase of AS [56]. ICAM-1 and VCAM-1, which belong to the immunoglobulin superfamily, are the crucial adhesion molecules involved in local inflammatory responses occurring in vascular walls [57–59]. ICAM-1 and VCAM-1 levels, which are predictors of acute coronary events, are associated with an increased cardiovascular risk. At early stages of vascular inflammation, VCAM-1 and ICAM-1 are upregulated in ECs to facilitate leukocyte adhesion to activated ECs and eventually promote endothelial dysfunction [60]. Our study clearly demonstrates that CLIC1 inhibition reduces the expression of ICAM-1 and VCAM-1. In addition, the inhibitory effects on adhesion molecules may offer a potential application of CLIC1 inhibition for the treatment of cardiovascular disorders, such as AS [61].

CLIC1, which is a protein in the GSH S transferase fold family, shows redox- and pH-dependent membrane association and Cl^−^ channel activity [28]. Coincidentally, the translocation of CLIC1 to the cell membrane under oxidative stress was observed in our in vitro experiments. By contrast, CLIC1 was not reconstituted into the phospholipid membranes in IAA94-treated HUVECs. We surmise that a specific mechanism facilitates the movement of CLIC1 into the membrane; moreover, Cl^−^ current plays an important role in AS. Soluble CLIC1 can directly integrate into preformed phospholipid vesicles where it functions as an anion-selective channel [62]. High intracellular Cl^−^ concentration was observed under oxidation compared with IAA-94 pre-treated or CLIC1^−/−^ cells. Although the Cl^−^ concentration has changed in this study, the mechanism of how CLIC1 forms an anion-selective channel and the electrophysiological phenomenon in AS still requires further studies. However, whether oxidation represents a pathway independent of lipid and acidic pH and whether it regulates the cell cycle in the AS remain unknown. After all, CLIC1 is detected on the plasma membranes of cells in the G2/M phase, and inhibition of CLIC1 function prolongs the mean time of cell cycle during cell culture [18]. The interaction of CLIC1 protein with membranes has been found to be lipid-dependent, and studies have shown that different combinations of phospholipids and cholesterol result in different functional activities of the protein [14, 62]. Moreover, one important finding of our experiments is that the significant increase in cholesterol level is associated with CLIC1 overexpression in vivo. The importance of cholesterol in the auto-insertion of CLIC1 into the membrane has been confirmed by Langmuir monolayer film experiments [63]. Furthermore, lipid accumulation is involved in the development of AS within the walls of large arteries, and whether a relationship exists between CLIC1 and cholesterol in AS warrants further investigation.

Thus, this study defines the roles of CLIC1 in AS, including promotion of oxidative damage and inflammation in ECs as well as the consequent disruption of endothelial function. The migration of CLIC1 to the plasma membrane in response to changes in the redox state of the ECs might help to form active chloride-selective ion channels and anionic current and facilitate the development of AS. Further research is warranted to clarify this issue.

## Conclusions

CLIC1 plays an important role in AS by aggravating oxidative damage and inflammation in ECs, which is accompanied by the translocation of CLIC1 into the membrane. These results highlight a novel molecular mechanism underlying the atherogenic effect of CLIC1 in ECs. Therapeutic approaches for inhibiting CLIC1 production might be valuable to prevent or treat AS.
